# Mathematical Modeling and Experimental Study of Cutting Force for Cutting Hard and Brittle Materials in Fixed Abrasive Trepanning Drill

**DOI:** 10.3390/mi14061270

**Published:** 2023-06-19

**Authors:** Ruijiang Yu, Shujuan Li, Zhengkang Zou, Lie Liang

**Affiliations:** School of Mechanical and Precision Instrument Engineering, Xi’an University of Technology, Xi’an 710048, China; 1170211009@stu.xaut.edu.cn (R.Y.);

**Keywords:** abrasive, trepanning tool, cutting force, axial force, torque

## Abstract

Hard and brittle materials have excellent physical and mechanical performance, which are widely applied in the fields of microelectronics and optoelectronics. However, deep-hole machining of hard and brittle materials is very difficult and inefficient due to the high hardness and brittleness of these materials. To improve the quality and efficiency of deep-hole machining of hard and brittle materials, according to the brittle crack fracture removal mechanism of hard and brittle materials and the cutting model of the trepanning cutter, an analytical cutting force prediction model of hard and brittle materials processed using a trepanning cutter is established. This experimental study of K9 optical glass machining shows that as the feeding rate increase, the cutting force increase, and as the spindle speed increase, the cutting force decrease. By comparing and verifying the theoretical and experimental values, the average errors of axial force and torque are 5.0% and 6.7%, respectively, and the maximum error is 14.9%. This paper analyzes the reasons for the errors. The results indicate that the cutting force theoretical model can be used to predict the axial force and torque of machining hard and brittle materials under the same conditions, which provides a theory for optimizing machining process parameters.

## 1. Introduction

Hard and brittle materials have been widely applied in aerospace, electronic equipment, and other fields due to their unique material performance, such as high hardness, high strength, low density, excellent chemical stability, etc. [1,2,3,4]. However, due to the characteristics of high hardness, high brittleness, and low fracture toughness, the removal mechanism of such materials is very different from the plastic removal method of metal materials, and they are typically difficult-to-machine materials. It is quite difficult to machine such materials with high precision, efficiency, and high quality, especially for deep-hole machining, which seriously restricts the application of hard and brittle materials in a wider range of fields [5,6,7]. Therefore, it is essential to conduct more deep research and improvements on the machining mechanism and deep-hole machining technology of hard and brittle materials.

At present, research on the removal mechanism of hard and brittle materials has made great progress. Lawn et al. [8] decomposed the complex elastic/plastic field beneath the sharp indenter into elastic and residual parts. Using indentation tests on soda glass and ceramics, they proposed a model for the propagation of median radial cracks when the sharp indenter contacts the workpiece. Marshall et al. [9] studied the mechanical principle of transverse crack propagation caused by sharp indenters in the contact area, proposed that the main driving force for material fracture comes from the residual components in the elastic/plastic region during the unloading process of the indenter, and established a mathematical model for transverse crack propagation. Lichun et al. [10] established a new grinding force model based on the theory that grinding force is composed of chip formation force and friction force. The relationship between grinding force and process parameters for processing workpieces of different materials was investigated, and the ratio of tangential force and normal force to chip formation force and friction force was studied. Fang et al. [11,12] studied the brittle/plastic transition process of optical glass processing via scratch tests and gradually changing the cutting depth. Gu et al. [13] utilized BK7 optical glass to conduct single-abrasive and double-abrasive scratch tests and analyzed the influence of normal load and scratch spacing on scratch morphology, material removal amount, and scratch depth. It was found that under the brittle removal mode, the material removal amount is closely related to the abrasive spacing. Yang et al. [14,15] conducted multiple scratch experiments on microcrystalline glass with different scratch sequences using a nanoindentation instrument and found that the interaction between scratches has a significant impact on the material removal mechanism. The experimental and analytical results indicate that within the range of crack interaction, the maximum principal stress decreases with the increase in scratch spacing.

Based on extensive research on the removal mechanism of hard and brittle materials, there have been several significant research studies regarding deep-hole machining of hard and brittle materials. At present, the trepanning drill is the main cutter for processing large-diameter deep holes in hard and brittle materials. The trepanning processing technology is a deep-hole processing technology that saves energy and raw materials with high efficiency and quality. This technology has been widely used in the deep-hole processing of hard and brittle materials. Abdelkawy et al. [16] established a mathematical model for the normal force generated via rotary ultrasonic drilling (RUD) based on two fracture modes of material plasticity removal and brittleness removal. Through drilling experiments on soda glass, it was demonstrated that the normal force in drilling is influenced by feed rate, spindle rotation speed, ultrasonic amplitude, frequency, and cutter abrasive size. Li et al. [17,18] conducted experiments on rotary ultrasonic constant feed rate drilling of hard and brittle materials and studied the effects of parameters such as spindle speed, feed rate, and ultrasonic power on cutting force and hole collapse size during the machining process. Pei et al. [19] proposed a theoretical prediction model of material removal rate by analyzing the material removal mode of hard and brittle materials in rotary ultrasonic machining. Wang et al. [20] introduced the removal mechanism and cutting force model of brittle materials in rotary ultrasonic machining and studied the formation mechanism and suppression methods of surface damage. Ding et al. [21] conducted rotary ultrasonic machining (RUM) and conventional drilling (CD) tests with a diamond trepanning drill and compared the effects of two processes on the axial force, torque, hole quality, and drilling surface roughness. The research results indicate that the axial force and torque decrease with the increase in spindle speed, while the change is not significant with the increase in feed rate. Zheng et al. [22] conducted constant feed rate drilling experiments on Al_2_O_3_ and SiC materials using a diamond drill and studied the changes in axial force and hole wall surface microstructure during the drilling process. Table 1 compares previous research on the trepanning processing of hard and brittle materials.

The above achievements have conducted multiple studies on the characteristics and patterns of processing hard and brittle materials from both theoretical and experimental perspectives, providing an important theory for the production practice of hard and brittle materials. However, these studies have not fully analyzed all the cutting forces generated during the trepanning process, and the influence of friction on the abrasive particles during the machining process has not been taken into account in theoretical modeling. This paper not only studies the chip deformation force on abrasive particles but also investigates the influence of friction on abrasive particles. At the same time, friction factors of different chip fluids are introduced into the model, and a mathematical model of axial force and torque generated during the machining process is established. In addition, this paper fully considers the type of cutter, the characteristics of abrasive particles on the surface of the cutter, and the random distribution characteristics and establishes a cutter model that can be analyzed.

Cutting force is an important parameter that characterizes the machining process and is one of the essential physical quantities that reflect the machining state. The parameter of cutting force directly affects the machining state and surface quality of the workpiece. Therefore, predicting cutting force can not only reflect the interaction between the cutter and the workpiece but also predict the surface quality of the workpiece. In this paper, a cutting force analytical prediction model of a fixed abrasive trepanning drill for cutting hard and brittle materials is proposed by studying the brittle fracture removal mechanism of hard and brittle materials, and a trepanning cutter cutting model is established. The machining experiment of optical glass (K9) analyzed the influence of different process parameters on cutting force, which verified the validity and rationality of the analytical prediction model of cutting force. The research results can provide theoretical guidance for the selection of process parameters and cutters for machining hard and brittle materials.

## 2. Materials and Methods

### 2.1. Research on Material Removal Mechanism

**Figure 1:**
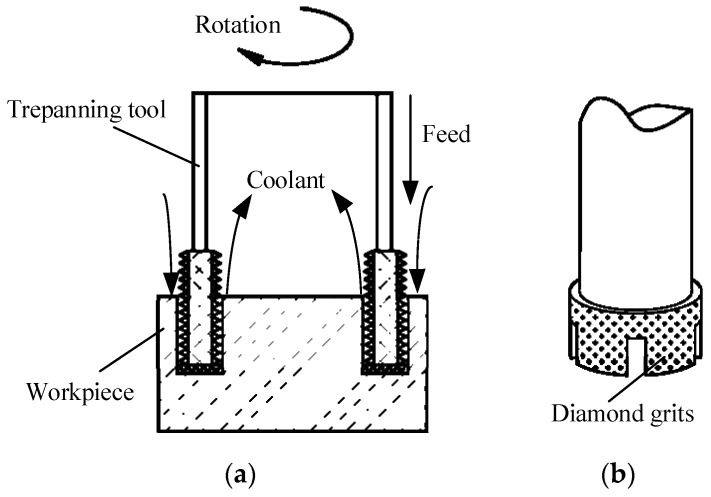
The schematic of trepanning processing. (**a**) Schematic of the processing. (**b**) Tool.

Fixed abrasive trepanning machining is a complex machining technology combining the material removal methods of traditional deep-hole drilling and grinding. The process of fixed abrasive trepanning machining is shown in Figure 1. The cutter with diamond particles is feeding towards the workpiece at a constant velocity or pressure while rotating at high speed.

Due to the distribution of a large number of irregularly shaped diamond abrasive particles with different protrusion heights on the end face of the trepanning cutter, when the diamond abrasive particles act on the workpiece, they come into contact with the working surface like small indenters, pressing into the workpiece surface to form cutting depth. Meanwhile, they scratch, erode, and scrap circumferentially with high velocity to the workpiece surface as the cutter rotates. Therefore, the removal mechanism of hard and brittle materials during trepanning machining can be considered as the high-velocity circumferential scratching of diamond abrasive particles while pressing into the workpiece, which is very similar to the indentation fracture mechanics used to study the pressing of hard and brittle materials by sharp indenters. According to the indentation fracture mechanics, the removal methods of brittle materials include plastic (ductile) shear and brittle collapse. When the cutting depth of the abrasive particles is less than the critical cutting depth of the material, the material is removed using the plastic shear of the abrasive particles, while forming smoother, crack-free scratches on the surface of the processing materials. As the cutting depth exceeds the critical cutting depth of the material, the bottom of the plastic deformation area of the pointed part of abrasive material produces median cracks and lateral cracks. The median cracks extend to the inside of the workpiece, while the lateral cracks, accompanied by the decrease in the abrasive pressure, extend parallel from the surface of the material to both sides and finally extend to the free surface of the material, fragment the material wrapped inside to form particles. Meanwhile, a concave is left on the surface of the material, which means that the material is removed in a brittle crushing way.

In this paper, the removal of materials is mainly based on brittle fracture removal by observing the surface morphology of the processed holes. To simplify the establishment and calculation of the analytical model, it is assumed that the material is completely removed via brittle fracture.

### 2.2. Establishment of Trepanning Cutter Model

**Figure 2:**
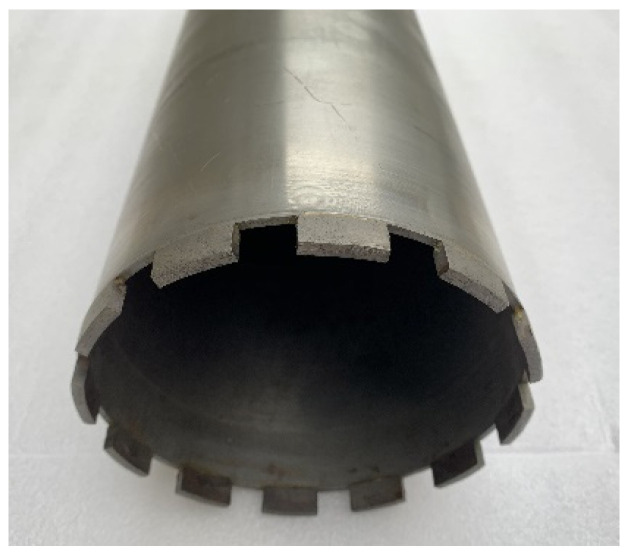
Trepanning tool.

The fixed abrasive trepanning cutter is a typical cutter for deep-hole machining of hard and brittle materials, which is welded from thin-walled stainless steel pipes and a cutter head with fixed abrasive particles, as shown in Figure 2.

**Figure 3:**
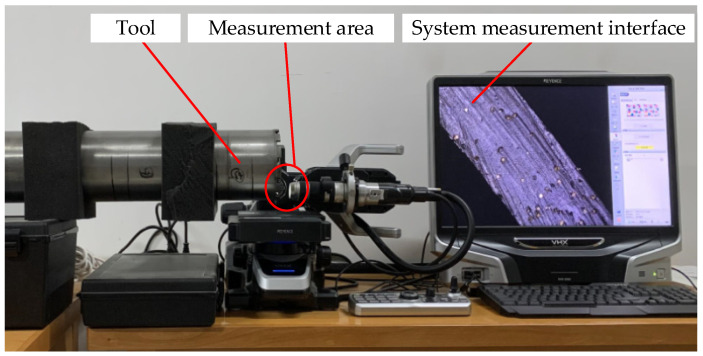
Observation process of abrasive grains on the tool surface.

To accurately study the distribution of abrasive particles on the end face of the trepanning cutter, the VHX-5000 3D microscope system with super wide depth of field produced by Keyence Company is used to observe the morphology of the end face of the trepanning cutter, as shown in Figure 3.

**Figure 4:**
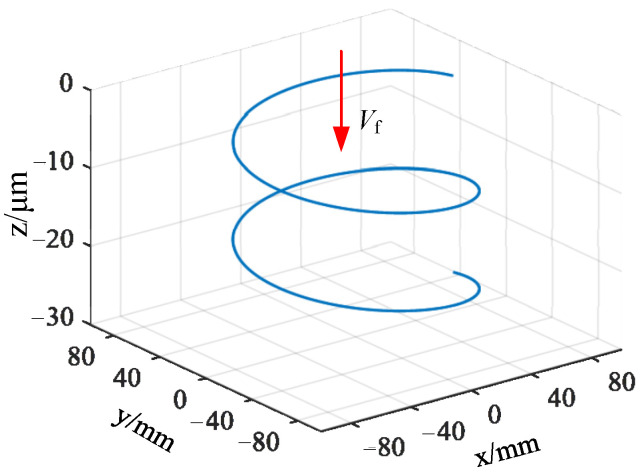
Abrasive grain equivalent motion path.

It can be seen that the diamond abrasive particles are randomly distributed on the end face of the trepanning cutter, and there are significant differences in the shape, protrusion height, and distribution density of the abrasive particles. Meanwhile, during the machining of fixed abrasive cutter, the path of the abrasive particles on the surface of the cutter tooth is different from the path of the abrasive particles on a traditional emery cutter. Its motion trajectory can be considered as the composition of the rotational motion of the cutter and the feed motion of the workpiece, resulting in a spiral trajectory with one pitch of feed rate, as shown in Figure 4. To establish an analytical model of the cutting force of the trepanning cutter, it is necessary to analyze and model the machining of the trepanning cutter.

To analyze the force on each abrasive particle, due to the distribution of abrasive particles on the end face of the trepanning cutter being random and uneven, it is necessary to simplify and regularly arrange the complex and irregular distribution of abrasive particles on the end face of the trepanning cutter and establish a theoretical geometric model of the trepanning cutter, to analyze the force state of the trepanning cutter. Then, the cutting force of a single abrasive particle is obtained by analyzing the material removal mechanism of a single diamond abrasive particle in machining process. The total cutting force of the cutter is based on all abrasive particles participating in grinding on the end face of cutter.

According to the cutting mechanism and the track of abrasive particles in the machining process, to establish the theoretical geometric model of the trepanning cutter, the following assumptions and simplifications for the abrasive particles on the end face of the trepanning cutter are made:

- All diamond abrasive particles participating in effective grinding have rigid cones with the same diameter;
- The distribution of effectively diamond abrasive particles on the end face of the trepanning cutter is generally even, which means that there is the same number of effective grinding abrasive particles per unit area;
- All effective diamond abrasive particles are continuously participating in grinding and have the same cutting depth;
- All the cutting depths of effective grinding abrasive particles are greater than the critical cutting depth of brittle fracture of the workpiece material, which means that the workpiece is removed in a brittle crushing way.

**Figure 5:**
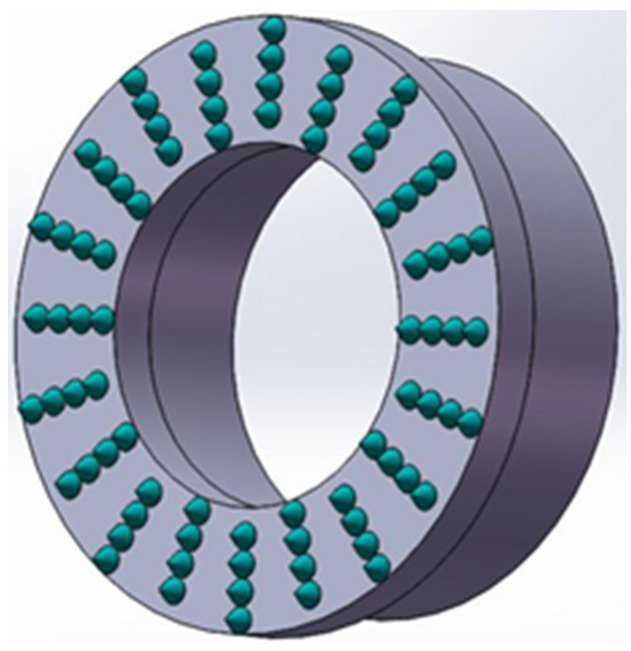
Equivalent model of tool end-face abrasive grains distribution.

Based on the above assumptions, take a toroidal cross-section with the difference of radius between the outer and inner circles, which is centered on the axis of the cutter arbor, on the end face of the trepanning cutter, and take difference of radius between the outer and inner circles as the cutting width of the abrasive particles. Each selected toroidal cross-section area with an equal number of effective abrasive particles is basically the same due to the turning radius of the trepanning cutter being much larger than the cutting width of a single abrasive particle. The toroidal cross-sections are arranged tightly in sequence to fully cover the end face of the trepanning cutter. The abrasive particles on each toroidal cross-section are aligned radially in sequence, which makes abrasive particles with the same radial direction into a set of cutting edges. The end face of the trepanning cutter is a cutting array composed of many sets of such cutting edges, as shown in Figure 5.

According to the theoretical geometric model of the trepanning cutter conducted above, the number of cutting edges on the end face of the trepanning cutter *Z* is expressed as follows:

(1) $$Z=\frac{N_{c}}{N_{0}}$$

where *N_c_* is the number of all abrasive particles on the end face of the trepanning cutter, and *N*_0_ is the number of abrasive particles required to form a set of cutting edges.

Due to the presence of many rectangular oil grooves at the front face of the trepanning cutter, as shown in Figure 2. These oil grooves result in a decrease in the proportion of the actual area of distributed abrasive particles on the end face of trepanning cutter, so a coefficient of cutter shape *G* is introduced to represent the percentage of the area of distributed abrasive particles. The number of all abrasive particles on the end face of the trepanning cutter *N*_c_ and the number of abrasive particles required *N*_0_ to form a set of cutting edges are expressed as follows:

(2) $$N_{c}=SC$$

(3) $$N_{0}=\frac{D-d}{2a}$$

(4) $$S=\frac{1}{4}\pi \left(D^{2}-d^{2}\right)G$$

where *S* is the end face area of the trepanning cutter (mm^2^), *D* is the outer diameter of the trepanning cutter (mm), *d* is the inner diameter of the trepanning cutter (mm), *G* is the cutter shape coefficient (%), *C* is the number of abrasive particles per unit area (1/mm^2^), and *a* is the cutting width of a single abrasive particle (mm).

Combining Equations (1)–(4) obtains the following equation:

(5) $$Z=\frac{1}{2}\pi a\left(D+d\right)CG$$

To establish the relationship between the cutting depth of a single abrasive and the feed rate, the feed rate of each cutting edge *a_f_* (mm) is expressed as follows:

(6) $$a_{f}=\frac{f}{Z}$$

where the feed rate per revolution of trepanning cutter *f* (mm) is

(7) $$f=\frac{60v_{f}}{n_{0}}$$

where *v_f_* is the workpiece feed rate (mm/s), and *n*_0_ is the rotation speed of trepanning cutter (r/min).

Combining Equations (5)–(7) obtains the following equation:

(8) $$a_{f}=\frac{60v_{f}}{n_{0}Z}=\frac{120v_{f}}{\pi n_{0}a\left(D+d\right)CG}$$

### 2.3. Force Analysis of Single Abrasive Particle

**Figure 6:**
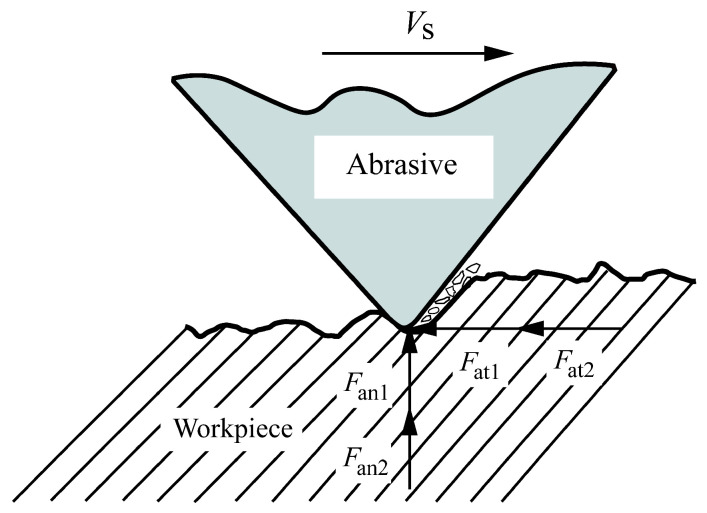
Force analysis diagram of a single abrasive grain.

Most prediction models for grinding force are based on analyzing the force acting on a single abrasive particle and then adding up the cutting forces of all the single abrasive particles involved in grinding to obtain the machining cutting force. This paper adopts a similar method to establish a mathematical prediction model for cutting force in trepanning machining of hard and brittle materials. In traditional plastic material processing models, the grinding force of a single abrasive particle is composed of two parts: chip deformation force and friction force. Chip deformation force can be divided into chip forming force and plowing force. However, plowing force is ignored in the grinding force model because it is much smaller than chip forming force. Therefore, according to the theory of indentation fracture mechanics, the force acting on a single abrasive particle during cutting can be decomposed into the normal force *F*_an_ (N) and the tangential force *F*_at_ (N). Based on the actual motion mode of diamond abrasive particles, a material removal process and cutting force model for single abrasive particle cutting hard and brittle materials are established, as shown in Figure 6.

The normal and tangential cutting forces acting on a single abrasive are expressed as follows:

(9) $$F_{\mathrm{an}}=F_{an1}+F_{an2}$$

(10) $$F_{\mathrm{at}}=F_{at1}+F_{at2}$$

where *F*_an1_ is the normal force exerted by chip deformation on abrasive particles (N), *F*_an2_ is the normal component of frictional force acting on abrasive particles (N), *F*_at1_ is the tangential force exerted by chip deformation on abrasive particles (N), and *F*_at2_ is the tangential component of frictional force acting on abrasive particles (N).

**Figure 7:**
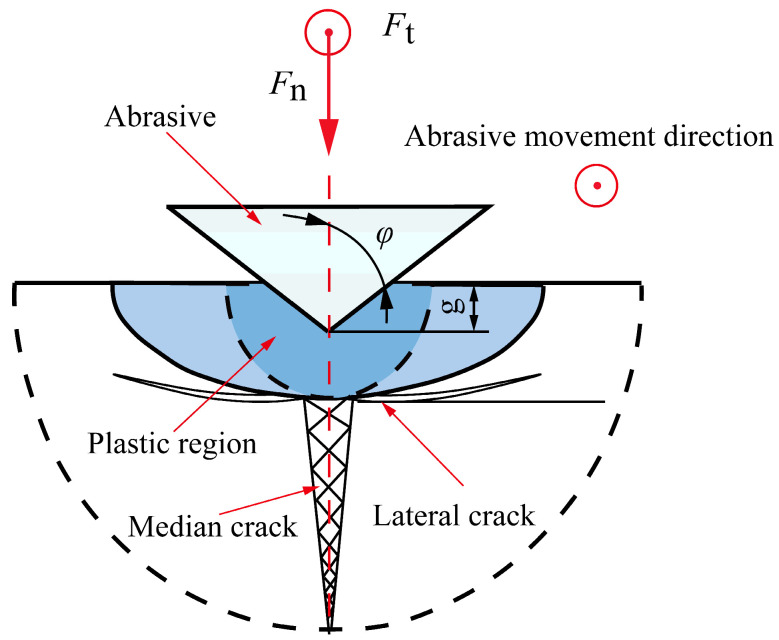
Single abrasive cutting crack system model.

According to the fracture mechanics principle of brittle materials, when abrasive particles cut brittle materials, the material removal mode is determined by the cutting depth. The cutting depth at which the material removal method changes is usually regarded as the critical cutting depth. When the cutting depth is less than the critical cutting depth of the workpiece material, the material is removed in a plastic mode. When the cutting depth is greater than the critical cutting depth of the workpiece material, the material is removed in a brittle mode. Figure 7 shows the crack situation generated when the material is in brittle removal mode.

**Figure 8:**
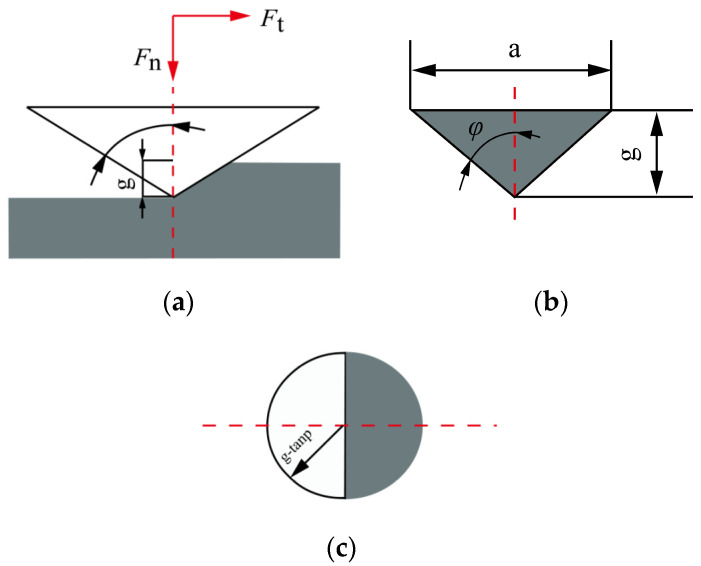
Schematic diagram of single abrasive contact surface projection: (**a**) front view, (**b**) left view, and (**c**) top view.

In the process of cutting hard and brittle materials with abrasive particles, the normal and tangential forces acting on the abrasive particles can be approximately calculated by multiplying the projected area of the contact surface between the abrasive particles and the workpiece in their respective directions by the material hardness. The projected area of the contact surface between the abrasive particles is shown in Figure 8.

By calculating the projected areas of abrasive particles in both directions, the normal force *F*_an1_ and tangential force *F*_at1_ acting on a single abrasive particle [23,24] are expressed as follows:

(11) $$F_{an1}=\frac{1}{2}\pi {\left(g\cdot \tan\varphi \right)}^{2}\cdot H$$

(12) $$F_{at1}=g^{2}\cdot \tan\varphi \cdot H$$

where *g* is the cutting depth of a single abrasive particle (mm), *φ* is the half angle of abrasive tip (°), and *H* is the hardness of the material (GPa).

Meanwhile, the relationship between the cutting width and depth of a single abrasive particle shown in Figure 8 is expressed as follows

(13) a=2tan φ⋅g

The cutting depth of a single abrasive particle is equal to the feed rate of each cutting edge.

(14) $$g=a_{f}$$

Combining Equations (8) and (11)–(14) obtains the following equations:

(15) $$F_{an1}=\frac{30v_{f}\cdot \tan\;\varphi \cdot H}{n_{0}\left(D+d\right)CG}$$

(16) $$F_{at1}=\frac{60v_{f}\cdot H}{\pi n_{0}\left(D+d\right)CG}$$

In the processing model of hard and brittle materials, due to the transverse crack extending to the free surface of the material, and the material wrapped by it breaks in advance or forms crack damage, there is discontinuous contact or irregular contact surface on the tangential processing surface in contact with the abrasive particles, so the friction force formed on the tangential contact surface is ignored, and only the friction force formed on the normal contact surface of the abrasive particles is considered.

The normal and tangential forces generated by a single abrasive particle under frictional force are expressed as follows:

(17) $$F_{an2}=0$$

(18) $$F_{at2}=\mu F_{an1}=\frac{30v_{f}\cdot \tan\;\varphi \cdot H\cdot \mu }{n_{0}\left(D+d\right)CG}$$

where *μ* is the friction coefficient.

Combining Equations (9), (10), and (15)–(18) obtains the following equations:

(19) $$F_{an}=\frac{30v_{f}\cdot \tan\varphi \cdot H}{n_{0}\left(D+d\right)CG}$$

(20) $$F_{at}=\frac{60v_{f}\cdot H}{\pi n_{0}\left(D+d\right)CG}+\frac{30v_{f}\cdot \tan\;\varphi \cdot H\cdot \mu }{n_{0}\left(D+d\right)CG}$$

### 2.4. Establishment of Cutting Force Model in Trepanning Processing

By analyzing the force situation of the trepanning drill, the normal force of all single abrasive particles participating in grinding is combined to form the axial force *F*_n_ of the cutter (N) and the tangential force of all single abrasive particles participating in grinding is combined to form the torque *M* of the cutter (N·m). Based on the above theory, the axial force and torque exerted on the cutter during the trepanning process are expressed as follows:

(21) $$F_{n}=dF_{an}=F_{an}\cdot SC=\frac{15\pi \cdot v_{f}\cdot \tan\;\varphi \cdot \left(D-d\right)\cdot H}{2n_{0}}$$

(22) $$M=dF_{at}\cdot r=F_{at}\cdot SC\cdot r=\frac{15v_{f}\cdot \left(D^{2}-d^{2}\right)\cdot H}{4n_{0}}(1+\frac{\pi \cdot \tan\;\varphi \cdot \mu }{2})$$

where *r* is the cutter radius of gyration (mm).

According to the above two equations, the cutting force generated during the trepanning process is affected by the workpiece feed rate *v_f_*, spindle speed *n*_0_, outer diameter of the trepanning cutter *D*, inner diameter of the trepanning cutter *d*, material hardness *H*, and half angle of the abrasive tip *φ* and friction coefficient *μ*. As the feeding rate increases, the cutting force increase, and as spindle speed increase, the cutting force decrease. In the subsequent experimental research, this study focused on the influence of cutter feed rate and spindle speed on cutting force.

## 3. Results

### 3.1. Test Conditions and Methods

**Figure 9:**
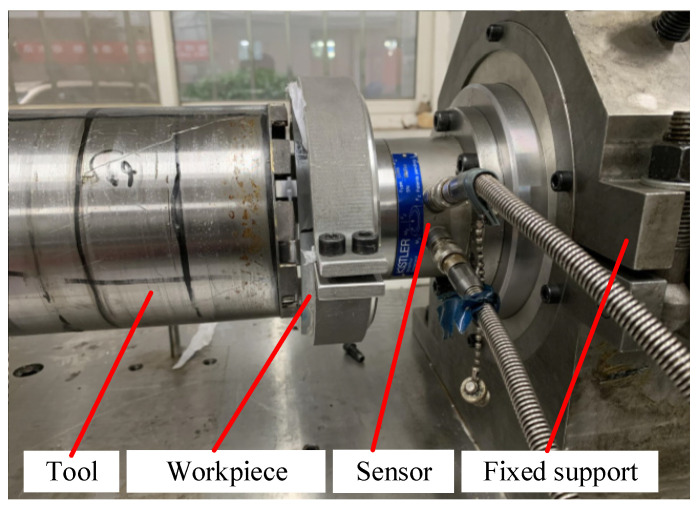
Sensor layout position and experimental site.

The experimental system is a deep-hole trepanning equipment developed and designed on the basis of the CW6163 ordinary lathe, which is mainly used for processing brittle material workpieces having a 150 mm diameter, with a maximum machining depth of 800 mm. The cutter used in the experiment having a 132 mm outer diameter and a 124 mm inner diameter is a fixed diamond deep-hole trepanning drill. The wall thickness of the cutter arbor is 2 mm, and the granularity of the diamond abrasive particles is 70/80. The material of the workpiece is optical K9 glass, and the size of the workpiece is *Φ*150 mm × 20 mm. The cutting fluid used in the experimental system is water. The measurement of cutting force in the experiment is completed by the Kistler 9721A binary force/torque sensor produced by Kistler Instrument Corporation and the WS-5921/U6 data acquisition instrument produced by Beijing Spectroscopy Company. In this experiment, the data collection frequency is 1000 Hz, and the cutting force is the average value of the collected cutting force during the machining process. The cutting force measurement system can simultaneously measure the axial force and torque acting on the cutter during the machining process. The experimental equipment and measurement scheme is shown in Figure 9.

To enhance the reliability of experimental data, take the average of two tests for each set of process parameters in the experiment, and the average was taken. The experimental processing parameters are given in Table 2.

### 3.2. The Influence of Process Parameters on Machining Cutting Force

**Figure 10:**
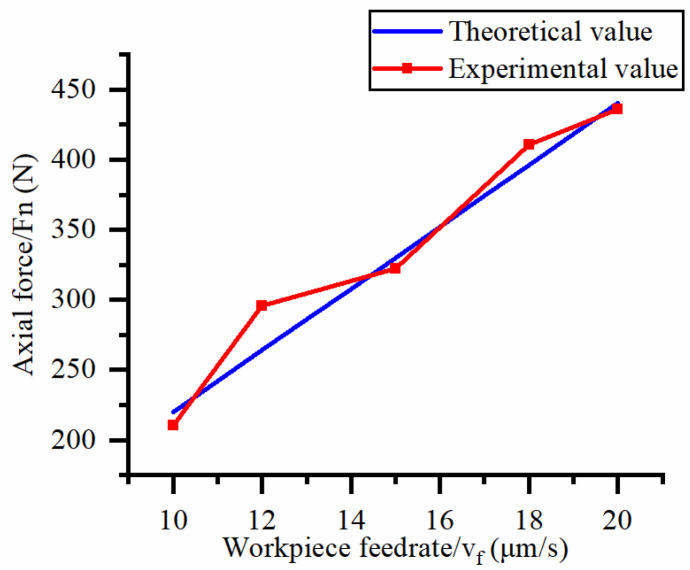
Influence of feed rate on the axial force.

**Figure 11:**
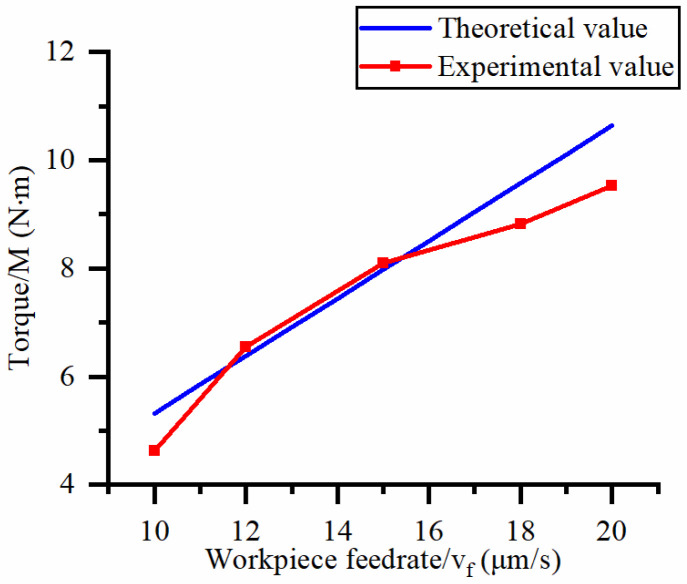
Influence of feed rate on torque.

**Figure 12:**
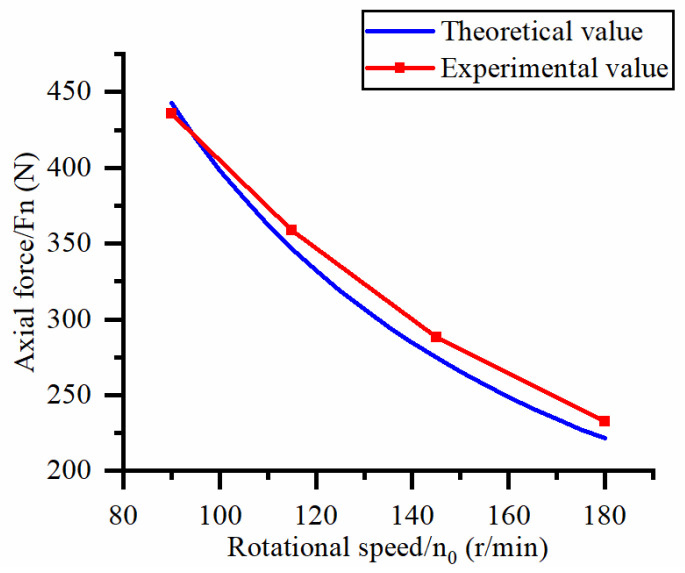
Influence of spindle rotational speed on axial force.

**Figure 13:**
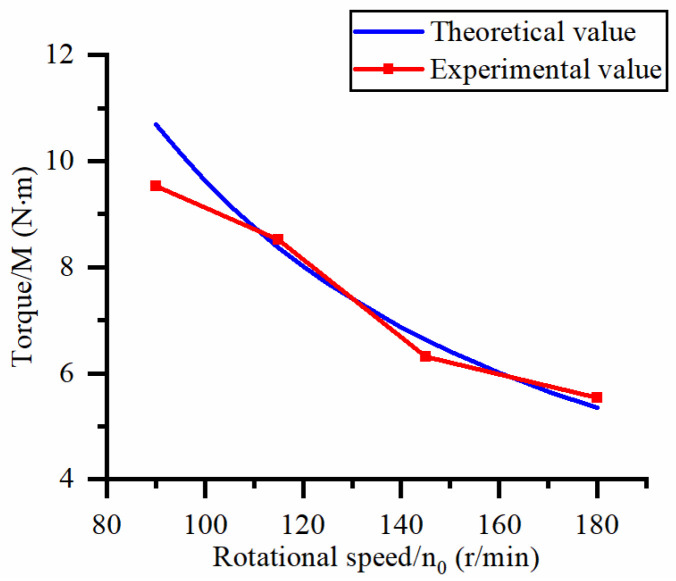
Influence of spindle rotational speed on torque.

This study focuses on exploring the influence of cutting process parameters (workpiece feed rate and cutter speed) on the machining cutting force of hard and brittle materials. Figure 10 shows the effect of different workpiece feed rates on the axial force at a spindle speed of 90.5 r/min. Figure 11 shows the effect of different workpiece feed rates on torque at a spindle speed of 90.5 r/min. Figure 12 shows the effect of different spindle speeds on the axial force when the workpiece feed rate is 20 μm/s. Figure 13 shows the effect of different spindle speeds on torque when the workpiece feed rate is 20 μm/s.

The axial force and torque increase with the increase in the workpiece feed rate, as shown in Figure 10 and Figure 11. By analyzing the slope changes of each curve in the two graphs, it can be seen that the axial force and torque exhibit a linear trend with the workpiece feed rate. Based on Figure 12 and Figure 13, the axial force and torque decrease as the spindle speed increases. By analyzing the slope changes of each curve in the two graphs, it can be seen that the decrease in cutting force gradually reduces as the spindle speed gradually increases, which indicates that axial force and torque are more sensitive to changes in spindle speed at high feed rates. Comparing the experimental results with the patterns shown in Equations (21) and (22), the results obtained by the two methods are basically consistent, thus verifying the correctness of the established mathematical prediction model for cutting force.

### 3.3. The Influence of Process Parameters on the Quality of Workpieces

Hole edge collapse and cracks are typical defects in the hole processing of hard and brittle materials. In the process of hole processing for hard and brittle materials, the quality and efficiency of hole processing are determined by the collapse during hole exit and hole entry. A smaller size of the collapse can improve the quality of hole processing and effectively reduce the cost of hole processing. The edge collapse situation of the processed workpiece during hole exit and hole entry is shown in Table 3.

From Table 3, it can be seen that there are varying degrees of edge chipping on the edges of the workpiece’s exit and entry holes, but the morphology of the two types of edge chipping varies greatly. The edge collapse at the entrance of the workpiece is mainly caused by small and continuous material breakage, with a maximum edge collapse size of about 0.5 mm. While on the exit edge of the hole, there was a large area of regional material cracking, with a maximum edge size of about 5 mm.

## 4. Discussion

To further validate the axial force model and torque model in Equations (21) and (22), the errors between the experimental and theoretical calculated values under different workpiece feed rates and spindle speeds are shown in Table 4.

According to calculations, the average error between the measured and predicted values of axial force and torque is 5.0% and 6.7%, respectively, with a maximum error of 14.9%. It has been proven that Equations (21) and (22) can be used to predict the axial force and torque of processing hard and brittle materials under the same conditions. The possible reason for the large torque error is that a large number of brittle fractures appear on the surface after abrasive processing, resulting in an uneven surface, which leads to changes in the calculation of the contact surface of a single abrasive in the theoretical model, and this calculation error has a greater impact on the tangential force of a single abrasive. In addition, the intersection of transverse cracks generated by adjacent abrasive particles affects the stress situation of a single abrasive particle, which also leads to a larger theoretical calculation value. Another possible reason for the error in the cutting force model is that the cutting edge of the cutter used in this experiment is very thin, and its turning radius is large. During the cutting process, the lateral vibration of the cutter causes an imbalance of the machining contact surface, which ultimately leads to experimental errors.

The hole edge collapse during hole exit and hole entry in the workpiece is mainly influenced by factors such as the size of the machining cutting force and the hardness of the workpiece material. Comparing the dimensions of the workpiece’s hole entry collapse under different process parameters, reducing the feed rate and increasing the spindle speed can improve the hole entry collapse situation, but there is no significant change in the hole exit collapse situation. The reason for this difference may be that reducing the feed rate and increasing the spindle speed reduces the cutting force of a single abrasive particle at the entry hole. While at the exit hole, due to the self-weight of the material core, the workpiece suddenly broke, and a large area of regional material cracking appeared at the hole mouth, resulting in no performance in the edge collapse caused by the feed rate and spindle speed.

## 5. Conclusions

(1) In this paper, the abrasive particles with complex and irregular distribution on the end face of the cutter are simplified and regularly arranged by analyzing the abrasive particle distribution on the surface of the cutter and the movement path of the abrasive particles, and the theoretical geometric modeling of the trepanning cutter is established.
(2) A force model of a single abrasive particle is established by analyzing the chip deformation and the frictional force of a single abrasive particle. By utilizing the founded theoretical geometric modeling of the trepanning cutter and the force model of a single abrasive particle, the mathematical prediction model of the axial force and torque of the cutter is established.
(3) The experiment of drilling optical glass shows that the cutting force changes linearly and increases with the increase in feed rate. The cutting force decreases with the increase in spindle speed, and under high feed rate conditions, the cutting force is more sensitive to changes in spindle speed. Decreasing the feed rate and increasing the spindle speed can improve the edge collapse of the entry hole, but there is no significant change for the exit hole.
(4) The errors between the experimental and predicted values of axial force and torque are 5.0% and 6.7%, respectively. The uneven surface of abrasive cutting, the intersection of transverse cracks generated by adjacent abrasive particles, and the lateral vibration of the cutter are the main reasons for errors.

## Figures and Tables

**Table 1:** Previous research on trepanning processing of hard and brittle materials.

S.no.	Author(s)	Drilling Method	Research Method	Research Content
1	Abdelkawy et al. [16]	RUM	Theoretical modeling, experimental research.	Variation of axial force.
2	Li et al. [17,18]	RUM	Experimental research.	Cutting forces, material removal rates.
3	Pei et al. [19]	RUM	Theoretical modeling, experimental research.	Material removal rate.
4	Wang et al. [20]	RUM	Overview	Cutting force, damage formation mechanism.
5	Ding et al. [21]	RUM, CD.	Experimental research	Drilling force, torque, quality of hole exits, and surface roughness.
6	Zheng et al. [22]	Low-frequency axial vibration drilling, CD.	Experimental research	Variation of axial force, micromorphology of hole wall surface drilled.
7	Lv et al. [6]	RUM, CD.	Experimental research	Formation mechanisms of exit-chippings.

**Table 2:** Experimental parameters.

Experimental Parameters	Value				
Spindle speed *n*_0_/(r/min)	90.5	113.5	144	181	
Feed rate *v_f_*/(μm/s)	10	12	15	18	20

**Table 3:** The edge collapse situation of the processed workpiece.

Process Parameters	Spindle Speed: 90.5 r/min Feedrate: 10 μm/s	Spindle Speed: 90.5 r/min Feedrate: 20 μm/s	Spindle Speed: 181 r/min Feedrate: 20 μm/s
Processed workpiece	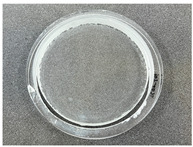	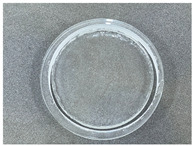	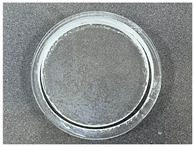
Entry hole edge collapse image	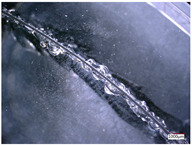	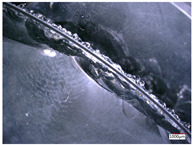	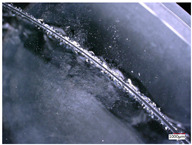
Exit hole edge collapse image	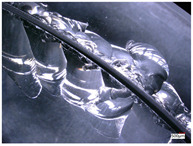	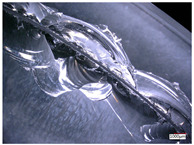	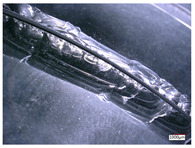

**Table 4:** Comparison of test results.

Serial Number	Spindle Speed *n*_0_/(r/min)	Feed Rate *v_f_*/(μm/s)	Test Value		Theoretical Value		Error (%)	
			*F*_n_/(N)	M/(N·m)	*F*_n_/(N)	M/(N·m)	*F*_n_	M
1	90.5	10	210.5	4.63	220.1	5.32	4.56	14.90
2	90.5	12	295.8	6.54	264.1	6.38	−10.72	−2.45
3	90.5	15	322.6	8.32	330.1	7.98	2.32	−3.86
4	90.5	18	410.6	8.82	396.1	9.57	−3.53	8.50
5	90.5	20	420.7	9.53	440.1	10.63	4.61	11.54
6	113.5	20	358.6	8.72	346.4	8.37	−3.40	−4.01
7	144	20	290.3	6.32	274.7	6.64	−5.37	5.06
8	181	20	232.6	5.54	221.3	5.35	−4.86	−3.43

## Data Availability

Not applicable.
